# Experiences of oral pre‐exposure prophylaxis use among heterosexual men accessing sexual and reproductive health services in South Africa: a qualitative study

**DOI:** 10.1002/jia2.26249

**Published:** 2024-05-02

**Authors:** Fatima Abegail Cholo, Siphokazi Dada, Catherine Elizabeth Martin, Saiqa Mullick

**Affiliations:** ^1^ Wits RHI University of the Witwatersrand Johannesburg South Africa

**Keywords:** HIV prevention, heterosexual men, pre‐exposure prophylaxis, oral PrEP, South Africa, qualitative study

## Abstract

**Introduction:**

South African men face a substantial burden of HIV and are less likely to test for HIV and initiate antiretroviral therapy if tested positive and more likely to die from AIDS‐related causes than women. In addition to condoms and circumcision, guidelines provide for the use of daily oral pre‐exposure prophylaxis (PrEP) as an HIV prevention intervention for any men who recognize their need and request PrEP. However, heterosexual men have not been a focus of PrEP programmes, and since its introduction, there is limited literature on PrEP use among men in South Africa. This study explores the experiences, motivators and barriers to oral PrEP use among heterosexual men accessing primary healthcare services in South Africa.

**Methods:**

This study forms part of a mixed‐methods implementation science study aimed at generating evidence for oral PrEP introduction and conducted in primary healthcare clinics in South Africa since 2018. Men aged ≥15 years who initiated oral PrEP and enrolled in a parent cohort study were purposefully invited to participate in an in‐depth interview (IDI). Between March 2020 and May 2022, 30 men participated in IDIs exploring their motivators for PrEP use, and experiences with accessing health services. Interviews were audio recorded, transcribed and analysed thematically.

**Results:**

The final analysis included 28 heterosexual men (18–56 years old). Motivations to initiate PrEP included fear of acquiring HIV, self‐perceived vulnerability to HIV and mistrust in relationships; health systems factors which motivated PrEP use included the influence of healthcare providers, educational materials and mobile services. Perceived reduction in HIV vulnerability and changing proximity to partners were reasons for PrEP discontinuation. Side effects, daily‐pill burden and stigma were noted as challenges to PrEP use. Health system barriers to PrEP use included limited PrEP availability, school and work demands, and inconsistent mobile clinic schedules.

**Conclusions:**

Our study reports on the experiences of heterosexual men accessing oral PrEP in real‐world settings and contributes to the limited literature among this population. We highlight multiple levels which could be strengthened to improve men's PrEP use, including individual support, education among partners and communities, and addressing health system barriers to access.

## 1 INTRODUCTION

South Africa had an estimated 7.6 million people living with HIV in 2022, of whom 2.6 million (34%) are men aged ≥15 years [1]. Despite the implementation of the “universal test and treat” programme in 2016, there were 150,000 new HIV acquisitions among people aged ≥15 years in 2022, with 52,000 attributed to men [1], implying that men face a substantial burden of HIV. Most HIV transmission in the country is through age‐disparate sexual partnerships (between younger women and older men) [2, 3], indicating that men form part of the HIV transmission cycle [4, 5] and cannot be neglected in HIV prevention efforts. Moreover, South African men are less likely than women to access health services, test for HIV, start antiretroviral therapy (ART) if tested positive and maintain viral suppression [5]. Not surprisingly, more South African men die from AIDS‐related causes than women [5].

Daily oral pre‐exposure prophylaxis (PrEP) has been recommended by the World Health Organization (WHO) as an additional HIV prevention method since 2012 [6]. Clinical trials have shown that oral PrEP is efficacious in reducing HIV by more than 90% when taken consistently [7]. In South Africa, PrEP has been available since 2016 [8], initially only to sex workers, with expansion to any population requiring HIV prevention in 2017 [9]. As of September 2023, 3579 sites offered oral PrEP, and between April 2020 and June 2023, 22% of 843,831 PrEP initiations in South Africa were in men [10]. While this indicates an interest in PrEP among men, the progress towards national PrEP initiation targets among men has fallen behind that of women [10].

Although the South African Department of Health (DoH) has recommended oral PrEP to anyone requiring HIV prevention, PrEP programmes have prioritized key populations, including men who have sex with men, sex workers, and adolescent girls and young women (AGYW) [11]. There is limited literature on heterosexual men's experiences of oral PrEP uptake and use in South Africa [12, 13]. Heterosexual men's experiences and motivators to PrEP uptake and use may be different to women [14]. Concluding solely from the experiences and perceptions of women may lead to challenges in understanding heterosexual men's PrEP needs and designing prevention programmes tailored to heterosexual men.

An early study looking at the perceptions of PrEP among heterosexual men indicates that most men are interested in initiating PrEP because of self‐perceived vulnerability to HIV, fear of acquiring HIV and mistrust of their sexual partners [13]. However, the study describes hypothetical views of non‐PrEP users. Motivation to PrEP use may evolve with experience, social context and time [15]; thus, understanding actual PrEP users’ experiences in real‐world settings is crucial in informing PrEP implementation and the success of PrEP. Additionally, the changing landscape of PrEP options requires us to better understand the individual and contextual factors which may influence men's PrEP use.

This study aims to shed light on men's experiences of oral PrEP use, detailing the motivators and barriers to using oral PrEP among heterosexual men accessing primary healthcare services in South Africa. Our study contributes to the scarce literature on oral PrEP use among heterosexual men.

## 2 METHODS

### 2.1 Study design and setting

This descriptive qualitative study is nested within Wits RHI's Project PrEP, a Unitaid‐funded implementation science study initiated in December 2018 and aimed at generating evidence to inform the introduction and integration of oral PrEP within a combination of HIV prevention and sexual and reproductive health (SRH) services in South Africa. Project PrEP is implemented in four distinct (urban, peri‐urban and rural) geographical settings in three provinces in South Africa: Mthatha and Gqeberha in the Eastern Cape, eThekwini in KwaZulu‐Natal and Tshwane in Gauteng. Study sites in each area consist of two fixed DoH primary healthcare clinics and a roving mobile clinic. We selected study sites in collaboration with the National DoH and provincial and district DoH officials, focusing on areas of highest priority for HIV prevention.

Study sites offer an integrated HIV prevention and SRH service package within the framework of adolescent and youth‐friendly services, including HIV testing, condom provision and oral PrEP. Although Project PrEP's demand generation activities focused on reaching and engaging AGYW (15–24 years) in HIV prevention services, older women and men were able to access the same health services, in accordance with national guidelines. There were, however, no specific engagement or demand‐generation activities undertaken which focused on men. Since oral PrEP was rolled out to include primary healthcare clinics in South Africa in 2020 [16], the experiences of men in this study are based at selected facilities that had implemented oral PrEP within the study.

### 2.2 Sampling

There were 28,903 clients initiated on oral PrEP between January 2019 and October 2022, of whom 4169 (14%) were men. Among those initiated on oral PrEP, 19,462 (67%) were initiated in fixed facilities and 9441 (33%) at mobile clinics. Of clients initiated on oral PrEP, 1810/24,734 (7%) women and 532/4,169 (13%) men enrolled in a nested longitudinal cohort. A subset of participants enrolled in the cohort was purposively selected and invited to participate in an in‐depth interview (IDI) if they met the following inclusion criteria: male, aged ≥15 years, initiated oral PrEP at one of the study sites, living or working in the study catchment areas, and willing and able to provide written informed consent. Eligible males were contacted telephonically or at a clinic follow‐up visit and invited to participate in an IDI at an agreed date and time. Participants were recruited to gain diverse views from men from different settings, including those who had consistently used PrEP and those who had interrupted or stopped PrEP. Recruitment continued until data saturation was reached.

### 2.3 Data collection

Trained male fieldworkers conducted interviews between March 2020 and May 2022 in the participant's preferred language (English, isiZulu, isiXhosa or Setswana). The interviews were conducted face‐to‐face and telephonically to accommodate participants who could not travel to the study sites due to the COVID‐19 pandemic and lockdown restrictions imposed in South Africa in 2020. A semi‐structured interview guide was developed to facilitate the discussion and to explore participants’ motivators of PrEP initiation and use, experiences with accessing health services and using PrEP. Interviews were conducted in a private space and lasted between 17 and 73 minutes. All interviews were audio‐recorded, and written consent was obtained prior to recording the discussion.

### 2.4 Data analysis

We conducted IDIs with 30 men across four geographical settings. Since this analysis focused on heterosexual men, data from two participants who self‐identified as non‐heterosexual were excluded from the analysis, leaving a total of 28 men in the final analysis. The analysis team consisted of two qualitative Researchers (FAC and SD), an Assistant Researcher (MM) and a medically trained HIV Technical Specialist (CEM). FAC and MM reviewed all the transcripts for accuracy. Data analysis was conducted inductively using NVivo 12, following six phases of thematic analysis: (i) familiarization with the data; (ii) generating initial codes; (iii) searching for themes; (iv) reviewing themes; (v) defining and naming themes; and (vi) writing up the findings [17].

To ensure the trustworthiness and rigour of the study, we employed the following quality criteria: *credibility*—we ensured credibility by using researcher triangulation, whereby the two qualitative researchers coded and analysed the data separately to provide different interpretations of the data [18]; *transferability*—we provided detailed descriptions with verbatim participants’ quotes in the findings [19]; *dependability*—we provided a dense description of the step‐by‐step techniques and data collection and analysis methods used by the study; and *confirmability*—data and interpretations of the study findings come purely from the participants’ perspectives and meaning of their experiences.

### 2.5 Ethical considerations

This study was approved by the Human Research Ethics Committees at the University of the Witwatersrand (M180860) and by the WHO Ethics Research Committee (Wits PrEP‐AGYW‐Main protocol 0003088). In addition, provincial health research ethics committees provided approvals to conduct the study at their health facilities. Written informed consent was obtained from all participants before conducting any study procedures. Participants received 100 ZAR (5,35 USD) for participating in the IDI and 50 ZAR (3 USD) for completing a study survey in the parent cohort study.

## 3 RESULTS

Table 1 presents the participants’ socio‐demographic characteristics. The final analysis included 28 men (with a median age of 27 years; inter quartile range [IQR]: 24–31.5), and most participants were ≤25 years (*n* = 17; 61%). The majority had initiated PrEP at a fixed facility (*n* = 23; 82%), 18 (67%) were employed and five (18%) reported being in school or tertiary education. Fourteen percent (*n* = 4) reported early sexual debut (≤14 years). Almost all (*n* = 26; 93%) were currently in relationships, with 11 (42%) participants having multiple sexual partners. Among those in relationships, nine (35%) reported having a partner living with HIV. Half (*n* = 14; 50%) of the participants reported engaging in transactional sex (i.e. receiving money or goods in exchange for sex), 13 (46%) reported sex under the influence of drugs or alcohol and 20 (71%) reported inconsistent condom use, in the last 3 months. Eighteen (64%) participants used PrEP consistently, seven (25%) had interrupted or stopped PrEP, and among three participants (11%), the pattern of PrEP use was unknown.

**Table 1:** Socio‐demographic characteristics of the study participants

Socio‐demographic characteristics	Total
	*N* = 28 (100%)
Age group	
18–24 years	11 (39.3%)
≥25 years	17 (60.7%)
Median age (IQR [p25–p75]) (years)	27 (24–31.5)
PrEP initiation site	
Fixed facility	23 (82.1%)
Mobile facility	5 (17.9%)
Currently in school or tertiary education	5 (17.9%)
Employment status	
Employed	18 (64.3%)
Unemployed	9 (32.1%)
Unknown	1 (3.6%)
Sexual debut	
13–14 years	4 (14.3%)
15–17 years	15 (53.6%)
18–23 years	8 (28.6%)
Unknown	1 (3.6%)
Relationship status	
In a relationship	26 (92.9%)
Single, no partner	2 (7.1%)
In relationship and have additional partner(s)	11 (42.3%)
In relationship and have a partner with HIV	9 (34.6%)
Transactional sex (in the last 3 months)	14 (50.0%)
Had sex under the influence of alcohol or drugs (in the last 3 months)	13 (46.4%)
Inconsistent condom use (in the last 3 months)	20 (71.4%)

Four main themes emerged: (1) motivators to initiate and use PrEP; (2) barriers to PrEP use; (3) reasons for PrEP discontinuation; and (4) challenges of PrEP use.

### 3.1 Motivators to initiate and use PrEP

#### 3.1.1 Fear of acquiring HIV

Participants felt anxious about contracting HIV and thus sought PrEP for their own protection. This fear of contracting HIV was triggered by learning about high HIV prevalence rates in the country and the impact of living with HIV.

“There are people who have the virus around me… they don't look fine. So, it came to my mind that if I get HIV I may also get that sick…” (21‐year‐old, KwaZulu‐Natal)

“When they say that the number of those infected with HIV is higher than 2000 here in South Africa, I saw that no I am also in danger. I also need to protect myself.” (27‐year‐old, Eastern Cape)

#### 3.1.2 Self‐perceived vulnerability to HIV

PrEP use was influenced by the desire to protect themselves from HIV, with participants expressing an understanding of sexual behaviours and relationships which may increase their vulnerability. These included being in serodifferent relationships, having multiple sexual partners, mistrusting their sexual partner, having unplanned casual sexual encounters, having condomless sex and alcohol use.

“…So, what motivated me the most [to start PrEP] is that I once found myself in a relationship with a person who has it [HIV]…” (32‐year‐old, KwaZulu‐Natal)

“…the thing that made me to take PrEP was that I found out that my girlfriend is HIV positive…” (28‐year‐old, Gauteng)

In the case where a participant was taking PrEP because he had multiple sexual partners, taking PrEP was an effort to protect himself and his primary sexual partner. The participants expressed guilt about putting their primary partners in situations where they may be vulnerable to acquiring HIV.

“What made me take PrEP is that sometimes as males, we do mess up… so I thought instead of just doing nothing, let me take PrEP for my safety's sake and to protect my partner from contracting HIV.” (28‐year‐old, Eastern Cape)

Some participants perceived themselves to be vulnerable due to their partners’ sexual behaviours. The participants suspected that their partners could have sexual partners outside their relationship where they may be exposed to HIV.

“…I might sleep with my partner while she is also sleeping with others outside… that is why I took PrEP because I can say I have a main chick, but I don't trust her…” (21‐year‐old, Eastern Cape)

Working away from home, from the main partner or not having a main partner led some participants to engage in unplanned casual sexual encounters. Often, they did not use a condom as it was not readily available because the sex was unplanned.

“…What made me to take this PrEP eish (laughs). [Eish is a colloquial exclamation used to express a range of emotions. In this context, it shows excitement.] Well, I'm currently single and then whenever I get a chance I just get intimate with certain people not a specific person…” (23‐year‐old, Gauteng)

#### 3.1.3 Attitudes towards condoms

Many participants reported engaging in condomless sex and using condoms inconsistently, despite not knowing their partner's HIV status. Reasons for inconsistent condom use/non‐use included maintaining relationships where partners do not want to use a condom and not enjoying sex when using a condom. As a result, the participants opted for PrEP as a better HIV prevention method.

“…it happened that the lady said that she gets hurt when we are using a protection then I saw that when I take PrEP there is hope.” (34‐year‐old, Eastern Cape)

“…When you're using a condom, it's not nice my brother. Also, your partner's reaction is not the same…” (21‐year‐old, Eastern Cape)

Some participants expressed perceptions that PrEP was a better HIV prevention method than condoms, particularly in the context of alcohol consumption where condoms may not be used due to forgetfulness or not being readily available.

“…condom is easy to forget maybe when you drink alcohol… So, when you are using PrEP, you know that even if mistake happens, of not using a condom, you feel safe.” (28‐year‐old, Eastern Cape)

“Condom is forgettable because we do drink, then you forget condom when you get home. Or you do not even have it in the house.” (27‐year‐old, KwaZulu‐Natal)

#### 3.1.4 Healthcare providers’ influence

Almost all participants mentioned encountering friendly healthcare providers (HCPs), in both fixed facilities and mobile clinics, who provided them with health information that was easy to understand.

“Here there is no difference. Because I take my pills here [mobile clinic] and I take them there [clinic], the nurse communicates and advises me to also use a condom…” (24‐year‐old, Eastern Cape)

“…I was very happy because they [nurses] made the situation very friendly, I was still not comfortable talking about HIV. They just made the whole environment an open environment and easy to talk about…” (23‐year‐old, Gauteng)

Some participants highlighted the positive roles the nurses played in their decision to initiate PrEP. When nurses provided comprehensive information in a positive manner, this helped the participants to understand and initiate PrEP.

“She [nurse] was very informative. In fact, if it wasn't for her, I don't think I would have considered taking the PrEP…” (28‐year‐old, Gauteng)

#### 3.1.5 Influence of information, education and communication materials

During the study, participants were provided information, education and communication (IEC) materials, including booklets and pamphlets about PrEP. The materials provided participants with in‐depth information, and as a result, they were motivated to access and initiate PrEP.

“…So, the booklets gave me more information about PrEP. How to use it, and the side effects and all that. So, it's because of that booklet that I eventually agreed to try the PrEP.” (28‐year‐old, Gauteng)

“I was called in the clinic to collect something, and I got them [IEC materials], read them, and I became interested. I came to test and then after testing I started.” (25‐year‐old, Eastern Cape)

#### 3.1.6 Mobile service access

Some participants accessed SRH services, including initiating oral PrEP and collecting monthly refills, at the roving mobile clinic which provided services in communities surrounding each primary healthcare clinic. Participants noted the convenience (in terms of distance), accessibility and visibility of the mobile clinics which motivated their PrEP uptake.

“…I thought it would be easier to have a mobile clinic than going to the clinic.” (23‐year‐old, Gauteng)

“The first time I started using PrEP, I accessed the services through the mobile truck. I was coming from work; I saw the mobile van and people were queuing… So, I got interested at that time, and it was my first day taking them (PrEP).” (35‐year‐old, Eastern Cape)

Mobile clinics were preferred by those who were concerned about the perceived stigma and privacy associated with fixed facilities. The mobile clinics were perceived to be not busy with less likelihood of being recognized by others who might question their visit and assume their HIV status.

“…we must use mobile cars…because you find that the mobile is not busy… But at the clinic when you come it is known that you are sick.” (40‐year‐old, KwaZulu‐Natal)

Additional benefits of mobile services included the absence of long queues, more time provided for the consultation and sharing information about HIV prevention strategies.

“I find it [service] good because we don't queue for long…” (21‐year‐old, Eastern Cape)

“Because when I take my pills here [mobile clinic] and I like the communication. The nurse is able to communicate and advises me that I should also use a condom.” (26‐year‐old, Eastern Cape)

### 3.2 Reasons for PrEP discontinuation

#### 3.2.1 Absence of a sexual partner

When participants were no longer sexually active or had no sexual partner, they perceived their vulnerability to HIV as low and discontinued PrEP.

“… I thought since I am no longer in that relationship. I found that because that has ended, I always test. So, I found that I don't have that thing [HIV].” (56‐year‐old, KwaZulu‐Natal)

#### 3.2.2 Changing proximity to partners

The PrEP journey of men who work away from home, from their sexual partners appeared to influence a start‐and‐stop pattern of use. The participants took PrEP daily when they knew they would be with their partners and discontinued PrEP when they departed from their partners.

“Maybe for around two weeks I had stopped. And within these two weeks you find that I don't have any; my girlfriend is not around…” (26‐year‐old, Eastern Cape)

### 3.3 Barriers to PrEP use

#### 3.3.1 Limited PrEP availability

Since oral PrEP was not yet widely available in primary healthcare clinics at the time of our study, the limited availability of PrEP in some facilities affected the participants’ PrEP use. The participants reported travelling home to areas where PrEP was not routinely offered in their local primary healthcare facilities. In most cases, these trips were extended, and when their PrEP pills were finished, they could not get refills and thus discontinued PrEP.

“I haven't been using PrEP for a long time… The reason why I wasn't using it [is] because I was in the village… but they do not have PrEP…” (21‐year‐old, KwaZulu‐Natal)

#### 3.3.2 School and work demands

Another barrier to oral PrEP use among those still in school and working included the inability to attend the clinic for follow‐up visits and PrEP refills due to school and work commitments, which resulted in PrEP discontinuation.

“I have stopped [PrEP] because of my job. That time, it was December. I didn't have a chance to be off, I was always working… So, that's why I didn't find time to come here.” (21‐year‐old, Eastern Cape)

#### 3.3.3 Inconsistent and unpredictable mobile clinic schedules

Participants who resided far from the fixed clinic relied on mobile clinics to access services, including PrEP. When mobile clinics do not arrive or do not stick to the schedule, participants are affected.

“So, by then I started taking PrEP that was late last year and then the schools were closed so that the mobile clinic was supposed to come back, but it didn't come back and then we were busy with exams I just went home and then that's when I stopped taking PrEP.” (23‐year‐old, Gauteng)

### 3.4 Challenges of PrEP use

#### 3.4.1 Experienced side effects

Most participants reported experiencing minor side effects during the first few days of PrEP use, including headache, dizziness, nausea, fatigue, weight gain, increased appetite and stomach cramps.

“…For those of us who don't take PrEP at times in the beginning, I would feel dizzy and I would get a headache. I gained weight and I didn't have energy to do anything…” (24‐year‐old, Eastern Cape)

However, being informed about the potential side effects during PrEP initiation prepared and equipped the participants to overcome the challenge of experiencing side effects without discontinuing PrEP.

“… But I already knew that if you are using PrEP, you will have a headache and you might feel dizzy. So, then I saw that okay these are the side effects they mentioned.” (28‐year‐old, Eastern Cape)

#### 3.4.2 Daily pill‐taking burden

Most participants struggled with taking daily PrEP pills consistently, mainly in the first month of PrEP use. Most reported forgetting to take PrEP daily, often triggered by being away from home, unplanned sleep over and alcohol consumption.

“The only challenge that I got was when I started to take PrEP. I used to skip a day.” (28‐year‐old, Gauteng)

“My challenge is that sometimes I would forget the time I am supposed to take it. Sometimes I would not be in the house by the time I am supposed to take it.” (25‐year‐old, Eastern Cape)

Some participants suggested that a longer‐acting pill or injectable PrEP would be better than the daily pill to alleviate the burden of daily pill‐taking and could further encourage PrEP uptake and use.

“But I think that they can maybe try the pill maybe can be an injection or a pill that can be taken once a month or maybe three months or it can be an injection for three months a lot of people would be motivated to come here and take PrEP…” (27‐year‐old, Eastern Cape)

#### 3.4.3 Stigma

The participants expressed HIV stigma associated with PrEP use by others in their communities. Oral PrEP is misidentified as an antiretroviral medication for HIV treatment rather than a prevention medication because it is taken daily and appears identical to HIV treatment tablets. Therefore, the participants reported skipping a dose when other people were around to avoid being mistakenly identified as living with HIV.

“You see the challenge comes when there is somewhere that I need to travel. And I can't just carry them [PrEP pills] with me and go with them. When I get there, I would just sleep at that place, but I left them at home…” (26‐year‐old, Eastern Cape)

When those around PrEP users constantly question PrEP, it can be challenging to continue using it.

“…sometimes you have to take it and there's people who say, “why are you always taking pills”. And you cannot even explain because they do not know it so, they won't understand…” (21‐year‐old, KwaZulu‐Natal)

Taking oral PrEP was also associated with negative connotations of having multiple sexual partners. Participants explained how disclosing to their partners led to suspicions of having sexual activity outside the relationship and thus resulted in conflict.

“…the challenge I had was my girlfriend, she had a problem with PrEP. She did not want it because she has this thing of saying I want to sleep with other females…” (35‐year‐old, Eastern Cape)

## 4 DISCUSSION

This study explored oral PrEP use and uptake among heterosexual men in real‐world settings in South Africa and contributes to the scarce literature on men and PrEP use. Consistent with other studies, participants’ motivation to access PrEP across various geographical settings was primarily driven by the desire to protect themselves from HIV, linked to a perception of heightened vulnerability to HIV [19, 20]. Participants perceived themselves to be vulnerable to HIV based on their sexual behaviour, which included having multiple sexual partners, being in serodifferent relationships, having unplanned sexual intercourse, and having condomless sex and challenges with condom use, particularly when using alcohol. For some participants, motivation to initiate PrEP centred on protecting partners and maintaining relationships, similar to a study among serodifferent couples [21]. In this study, PrEP was seen as a preferred HIV prevention method compared to consistent condom use, which was perceived to make sex less pleasurable and was challenging when sex occurred under the influence of alcohol; PrEP counselling should highlight the importance of condom use in addition to PrEP use to enhance protection against HIV and other sexually transmitted infections. Consistent with the literature, alcohol consumption motivated PrEP uptake in our study [12, 22]. However, we did not explore the potential impact of alcohol consumption on consistent PrEP use and retention in PrEP care, an area that may be explored in future studies.

Previous studies have found that negative provider attitudes, lack of privacy, long waiting times and HIV testing are substantial barriers to PrEP uptake and use [12, 22]. In our study, we found several health system factors which supported PrEP use and uptake. These included the positive influence of HCPs, information provided through IEC materials and the role of mobile clinics. In contrast with other studies, positive interactions with HCPs and their attitudes while sharing information about PrEP were reported in our study as a motivator for PrEP uptake [12, 23]. The HCPs in our study were trained and mentored to provide PrEP and youth‐friendly facility‐based improvements (i.e. youth waiting areas, youth time slots). In addition to an enhanced physical environment, the training and mentorship programmes could have contributed to improved HCP skills, and the positive experiences with HCPs reported by the participants. IEC materials were influential in improving the participants’ PrEP knowledge and encouraged PrEP uptake and use. In keeping with a previous South African study [24], this highlights the importance of accessible, accurate information about HIV prevention and PrEP services. Mobile services may provide an opportunity to address barriers to healthcare access, particularly among men [25]. Similar to previous studies, our study highlighted the benefits of mobile clinics such as convenience and service quality, while avoiding long waiting times at fixed clinics [23 ^,^ 26, 27]. In our study, mobile clinics minimized the need to travel long distances to access services, and as were not often too busy, minimized the perceived stigma, maintained privacy and allowed sufficient time for consultation. However, friendly HCPs were described at both mobile and fixed facilities. The role of decentralized service models (i.e. mobile clinics, pharmacies and community‐based services) in improving access and uptake of PrEP requires further research.

PrEP discontinuation is often driven by reduced or changing perception of vulnerability to HIV and prevention needs, such as when one is no longer sexually active, as has been reported in a study among heterosexual men [14]. PrEP counselling should include education on how to safely stop and restart PrEP given intermittent PrEP use, to ensure protection against HIV during periods of vulnerability. Our results highlight the unique structural barriers, including PrEP availability at local facilities, inconsistent mobile clinic schedules, travelling and not having time for PrEP refill visits because of work and school commitments, that may affect PrEP continuation in real‐world settings. Programmes offering PrEP to men could consider providing flexible services at locations and times that are more convenient to them.

Consistent with the literature, participants in our study experienced mild, temporary side effects when they started taking PrEP [14, 28]. However, in contrast to other studies, none of the participants in our study reported discontinuing PrEP due to side effects [14, 22, 24, 29]. Our study emphasizes that counselling and managing side effects are critical during PrEP initiation and as highlighted in our findings, provided reassurance, and may have prevented PrEP discontinuation. As in previous studies, men in our study often forgot to take their oral PrEP when away from home [30, 31, 32]. Unprompted, some men in our study explicitly expressed a preference for long‐acting oral or injectable PrEP. Preference for long‐acting PrEP methods, including long‐acting injectable cabotegravir, was also expressed in a previous study among heterosexual men [33]. Providing and integrating long‐acting PrEP methods to existing HIV prevention services will offer men additional choice and could address challenges to oral PrEP use [32, 33, 34].

Although individuals may intend to initiate and use PrEP, PrEP‐related stigma is a potential obstacle to effective use. Similar to previous studies, oral PrEP was perceived to be associated with negative connotations of having multiple sexual partners [14, 31]. The lack of PrEP availability in public primary clinics in rural areas and lack of awareness of its use led community members to confuse daily oral PrEP with ART, and PrEP users expressed being misidentified as living with HIV. Similar findings were observed in previous studies conducted in rural areas [13, 14, 35], and this stigma was associated with lower adherence [36, 37]. Therefore, there is a need to increase community education and awareness about HIV prevention and PrEP [13] and engaging men in this type of education.

## 5 Limitations

Our study focused only on the experiences of men who initiated oral PrEP. Thus, the lack of perspectives from men who declined oral PrEP limits our broader understanding of barriers to PrEP uptake among this population. Some respondents, however, offered “collective” perspectives on challenges to PrEP use denoted by their use of the words “us,” “we” and “males” in their responses—offering insights into the current barriers of PrEP use among men generally. Our study was conducted at a time when oral PrEP was just beginning to be introduced, but was not yet widely available in primary healthcare clinics, as well as during the period of the COVID‐19 pandemic and its associated lockdown restrictions. Our findings may, therefore, be reflective of early PrEP adopters, or those who had greater self‐motivation regarding their health and HIV prevention access. Like many qualitative studies, our study utilized a small sample size, and thus caution should be employed when transferring the findings beyond the current study. Although the sample size may have been a limitation, no new themes emerged in the final interviews, indicating that thematic saturation was reached.

## 6 CONCLUSIONS

Our study explored heterosexual men's experiences of oral PrEP uptake and use and contributes to the scarce literature on PrEP use among this population. Men's motivations for PrEP uptake and use were driven by their perceptions of vulnerability to HIV, fear of acquiring HIV, challenges using condoms, alcohol consumption and partnership mistrust. Health system factors including the influence of HCPs, IEC materials and mobile services supported PrEP use. Although men need HIV prevention services, factors including limited PrEP availability, school and work demands, and inconsistent mobile clinic schedules hinder PrEP use. Reported challenges to continued PrEP use included experiencing side effects, daily pill burden and stigma. Our findings highlight a need for a multifaceted approach to strengthen HIV prevention services for men, including individual support for PrEP and condom use, PrEP education among partners and communities to address ongoing stigma, responsive service delivery models which address health system barriers and access to long‐acting PrEP methods, to improve PrEP uptake and use among this population.

## COMPETING INTERESTS

The authors declare that they have no competing interests.

## AUTHORS’ CONTRIBUTIONS

CEM and SM contributed to the conception and design of the study. FAC reviewed the transcripts. FAC and SD coded and analysed the data. FAC and SD wrote the first draft of the manuscript. CEM and SM reviewed the manuscript. All authors contributed to the manuscript revision and read and approved the submitted version.

## FUNDING

This work was financially supported by Unitaid [grant number 2017‐21‐Wits‐PrEP].

## Data Availability

The data (transcripts) underlying the results presented in this manuscript are readily available. Requests to access the data should be directed to fcholo@wrhi.ac.za.
